# Lethal Doses of Saponins from *Quillaja saponaria* for Invasive Slug *Arion vulgaris* and Non-Target Organism *Enchytraeus albidus* (Olygochaeta: Enchytraeidae)

**DOI:** 10.3390/insects11110738

**Published:** 2020-10-28

**Authors:** Mantas Adomaitis, Grita Skujienė

**Affiliations:** Department of Zoology, Institute of Biosciences, Life Sciences Centre, Vilnius University, Saulėtekio av. 7, LT-10257 Vilnius, Lithuania; grita.skujiene@gf.vu.lt

**Keywords:** plant extract, molluscicide, resistance, *Arion*, *Enchytraeus*

## Abstract

**Simple Summary:**

The Spanish slug, known by its scientific name *Arion vulgaris*, causes significant damage to agriculture and private gardens around the world. To control this pest, the use of saponin-rich plant extracts is gaining importance as they exhibit strong molluscicidal activity against gastropods and are safe (as saponin residues) in agriculture products. However, despite their proven safety in food, they still do not have a widespread application due to their liquid form and the absence of more accurate knowledge of their effects on other organisms. In this study we evaluated an extract from the bark of the soap tree *Quillaja saponaria* on slugs and on the white worms *Enchytraeus albidus*. It was found that slugs were significantly more sensitive to saponin extract at 2 and −1 °C compared to 15 °C. The lethal effect of saponins was stronger on adult slugs than on juveniles. However, lethal effect on worms was much stronger than on slugs. Overall, our results show that *Q. saponaria* saponins may be a successful slug control tool, especially during colder periods, but its concentration should be selected according to the age of slugs. However, high toxicity to white worms limits its use as an environmentally benign alternative means of slug control.

**Abstract:**

The slug, *Arion vulgaris* Moquin-Tandon, 1855, is a serious pest in agriculture and private gardens. White worm, *Enchytraeus albidus* Henle, 1837, is an important model of decomposer organism in the terrestrial ecosystem. Saponins, which are secondary metabolites of plants, have previously been shown to have some molluscicidal effect. We investigated which doses of saponins are lethal to the slug, *A. vulgaris*, and to the non-target organism, *E. albidus*. An aqueous solution with different concentrations of saponin extract from the bark of the soap tree, *Quillaja saponaria* Mol., was used in repeat treatments. Slugs were tested in filter paper contact tests as they are naturally exposed to soil contact while crawling. Worms were tested in soil contact tests as they live below ground. It was found that lethality of saponins depends on the slug age group and the environmental temperature. The median lethal concentration (LC_50_, at 15 °C) on adults was 68.5 g/L, and on juveniles, 96.9 g/L. The slugs were significantly more sensitive at 2 and −1 °C compared to 15 °C. The LC_50_ (at 6 ℃) on *E. albidus* was 2.7 g/L (or 0.5 g/kg dry weight of soil), far below those in *A. vulgaris* (at 15 ℃ and lower). The LC_50_ for worms at -1℃ was also significantly lower than at 6 ℃. Therefore, we can conclude: (1) that *Q. saponaria* saponins may be a successful slug control tool used during colder times of the year, but its concentration should be selected according to the age group of *A. vulgaris*; (2) this measure is more toxic than expected to white worms, which limits its use.

## 1. Introduction

Slugs are important pests in a wide range of horticultural and arable crops [1,2] and the more frequent use of conservation tillage in agriculture is leading to an increase in slug pest problems around the world [3], especially when some of them are invasive species. The spread of invasive species constitutes one of the most important drivers for the global change of biodiversity and ecosystems [4]. The slug, *Arion vulgaris* Moquin-Tandon, 1855 (syn. *Arion lusitanicus* auct. non-Mabille, 1868), is an invasive species which has spread and become established in many European countries since the 1950s [5,6].

Because of the negative effects of common molluscicides in humans and other non-target organisms [7], new effective and environmentally friendly control methods are investigated. Some of them as mechanical barriers (diatomaceous earth, hydrated lime, sulfur, fumed silica, wood ash) are quite effective but insufficient in humid weather conditions when soil moisture adversely affects the efficacy of barrier materials [8,9]. Others, such as nematode *Phasmarhabditis hermaphrodita*, are commercialized for biocontrol applications, but have been shown to be less capable of killing adult *Arion* sp. [10,11] Bioactive compounds derived from different plant species are the new niche where new active ingredients for molluscicides are sought and even invasive plants can be used successfully [12].

Therefore previous studies have shown that saponin-rich plants exhibit strong molluscicidal activity against terrestrial gastropods [13] and are safe (as saponin residues) in food and agronomic products [14]. Saponins are the natural glucosides found in plants where they occur as secondary metabolites [15]. Due to their amphiphilic nature, saponins can enter the cell membrane and cause increased cell permeability, resulting in disordered circulation of water and ions and disruption of membranes [16]. In vitro studies show that saponins have strong anti-cholesterol and anti-carcinogenic properties [15,17]. The most recent data showed that saponin products help stimulate the immune response to co-administered antigens and have become increasingly important in the development of prophylactic and therapeutic vaccines [18].

Slotsbo et al. [19] reared slugs in captivity and established that slugs have the ability to survive temperatures around 0 °C and some individuals can survive even at −3 °C for time periods shorter than one day. This gives the idea that extending the time of using molluscicides in the cold periods of late autumn when young slugs are hatching, and in the spring when frosts are a common phenomenon but slugs are active, could be more effective for the control of slugs.

Our aim was to test whether saponins would be a sustainable alternative to current molluscicides in the control of the invasive slug pest, *A. vulgaris*, at different temperatures. We used saponins extracted from the bark of the tree, *Quillaja saponaria* Mol., and tested the effect on the target species, *A. vulgaris*, but we also wanted to determine its effect against non-target organisms. Therefore, we investigated the effect on an important decomposer in soil ecosystems, the white worm, *Enchytraeus albidus* Henle, 1837, known as one of the suitable models of bioindicators for soil health and quality [20,21].

## 2. Materials and Methods

### 2.1. Sampling and Maintenance of Invertebrates

Adult *A. vulgaris* slugs were collected from a wild population in Silkeborg, Denmark (latitude: 56°9′18″ N; longitude: 9°33′14″ E) in August–October 2014. Alive slugs were identified to species level with the use of identification charts [22]. After experiments slugs were dissected and species identification was confirmed in the form of distal genitalia [22]. Juveniles were reared from eggs produced by the field-collected slugs identified earlier.

*Enchytraeus albidus* worms were produced from a laboratory culture obtained in 2005 from the fish food company Tierfischfutter in Jena, Germany. The loamy sand used in all experiments was from a Danish pea field at Foulum near Viborg, and had the following composition: 35% coarse sand, 46% fine sand, 9.4% silt, 8.9% clay, and 1.7% organic matter with a pH of 6.8 [23].

The animals were maintained in containers of different sizes with lids and ventilation holes.

### 2.2. Molluscicidal Activity of Saponins on A. vulgaris Slugs

Saponin extract (QE) powder from the bark of the soap tree, *Quillaja saponaria*, (Sigma Aldrich, St. Louis, MO, USA, CAS No. S7900, 10%) was used. The powder was dissolved in distilled water to create a stock solution, which was used to prepare the different concentrations. The range of QE concentrations in water solution was based on pilot tests. Five and more replicates were prepared for each test concentration and control.

QE concentrations for slugs were: 10, 20, 25, 50, 100, and 150 g/L. All surfaces of each container and the Petri dish for slugs were lined with filter paper soaked with 1 mL of QE aqueous solution. The control treatment was distilled water.

A total of 670 slugs were used for these toxicological experiments in different temperatures (Table 1). Standard methods and durations selected by Iglesias et al. [24] and de Souza et al. [25] were used for maintaining and testing. The experiments were conducted under controlled environmental conditions in an incubator with a constant temperature and light–dark cycles of 12 h light and 12 h dark. The methods for keeping slugs in colder conditions were selected from Slotsbo et al. [19]

Adult and juvenile slugs were exposed by dermal contact for 48 h to cellulose filter paper soaked with QE solution at a temperature of 15 °C (Table 1). The survival rate was measured after 48 h. Adult *A. vulgaris* slugs were placed individually in plastic containers (7 cm diameter, 4.5 cm height) and juvenile slugs were placed in Petri dishes (9 cm diameter, 1.5 cm height) with five individuals in each dish. To assess the impact of temperature on the effectiveness of molluscicide at QE concentrations of 10, 20, and 50 g/L for adult slugs, low-temperature experiments were completed, one at 2 °C and the second at −1 °C according to Slotsbo et al. [19] Prior to the low temperature (at 2 °C and −1 °C) experiments, *A. vulgaris* slugs were cold acclimated by keeping them for one week at 10 °C, one week at 5 °C, and then for four weeks at 2 °C to induce freeze tolerance. Slugs were fed with carrots, lettuce, and pellets of dog food ad libitum. After acclimation, slugs were exposed to QE solution (of 10, 20, and 50 g/L) for two days at 2 ℃ and the survival rate was assessed. The freezing experiment at −1℃ was performed by removing slugs to clean containers with a water-moistened paper towel. A small piece of ice was added to initiate freezing, and the containers were moved to an incubator at −1 °C. After two days the containers at −1 °C were placed at 2 °C for 24 h to allow thawing. The survival of slugs was checked immediately after thawing.

### 2.3. Wormicidal Activity of Saponins on E. albidus Worms

The range of QE concentrations in water solution was based on pilot tests. Five and more replicates were prepared for each test concentration and control.

A total of 580 worms were used for toxicological experiments in different temperatures (Table 1). Standard methods and durations were used for maintaining and testing [26]. The experiments were conducted under controlled environmental conditions in an incubator with a constant temperature and light–dark cycles of 16 h light and 8 h dark.

QE concentrations for worms were 1, 2, 3, 4, and 5 g/L. Prior to use, the soil was dried for 24 h at 80 C, sieved through a 2 mm mesh and stored at room temperature until use. In the experiments, the solution of *Quillaja saponaria* aqueous extract content adjusted to 19% of dry soil weight (pF approximately 2, corresponding to approximately 50% of water holding capacity). These were homogeneously mixed. The soil was stored for one day before further use. The control treatment was distilled water.

Worms were placed individually in plastic containers (4.16 cm^2^) with 5 g, 3 cm height of moist soil, and food (50 mg of rolled oats). After one week, additional oats were added if the previous oats had been eaten. After 14 days, the survival of all the worms was assessed.

### 2.4. Statistical Analysis

Per experimental condition, datasets (*n* = 5) at survival were obtained. For every dataset pair-wise comparisons were performed between water controls and QE using student *t*-test (for normally distributed data) and the Mann–Whitney U test (for data with non-normal distribution). Each dataset was independently tested with the Kruskal–Wallis test for a comparison of surviving between *A. vulgaris* adults and juveniles, survival of slugs in different temperatures and survival of worms in different temperatures. The internal pair-wise comparisons identified significant differences with a 0.05 significance.

The Spearman correlation coefficient was used to examine the relationship between the concentrations of QE and the number of animals that survived.

Dose–response modelling and graphs were prepared using R Studio program, Drc package [27]. The LC_50_ value indicates the concentration of toxins that kill 50% of individuals in the sample. Dose–response data were described using a log-logistic model according to the Formula (1):

Y = d/(1 + exp^(b(log(x) − log(e))))(1)

where d refers to the upper and the lower horizontal asymptotes, e is the inflexion point corresponding to the LC50 value and b is the slope around e [27].

## 3. Results

### 3.1. Molluscicidal Activity of Saponins on A. vulgaris Slugs

**Figure 1:**
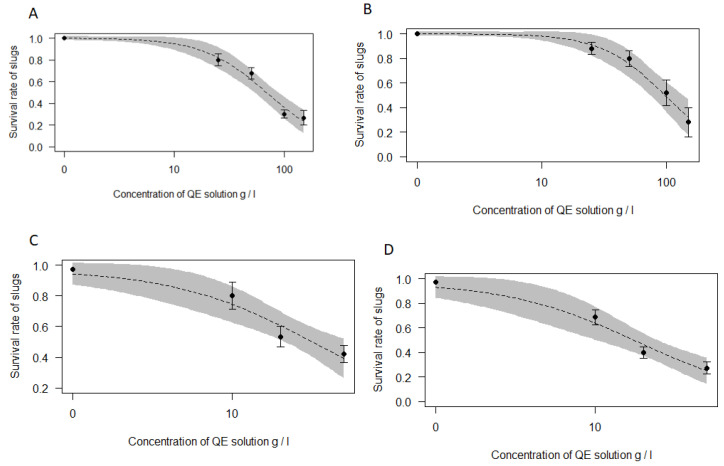
Dose–response curves for the toxicity of solution of *Quillaja saponaria* aqueous extract (QE) on: (**A**) *A. vulgaris* adults at 15 °C; (**B**) *A. vulgaris* juveniles at 15 °C; (**C**) *A. vulgaris* adults at 2 °C; (**D**) *A. vulgaris* adults at −1 °C. A grey band on the curve represents the standard error of the dose–response model. Black dots show the mean (with standard error bars) effect of the tested concentration of QE solution. Black dots represent tested concentrations of QE solution: 0; 25; 50; 100; 150 g/L (**A** and **B**) and 0; 10; 20; 50 g/L (**C** and **D**).

The effect of saponins from QE on *A. vulgaris* adults and juveniles exposed to four different doses for 48 h at 15 °C is illustrated in Figure 1A,B. The results show that molluscicidal activity of saponins on *A. vulgaris* adults and juveniles increased with the higher QE concentration.

We observed moderate negative Spearman correlations between QE concentration and survival of adult slugs (r_s_ = −0.51, *p* < 0.05; based on 125 slugs) and between QE concentration and survival of juveniles (r_s_ = −0.54, *p* < 0.05; based on 125 slugs). The survival of juveniles was significantly higher than the survival of adult slugs (Kruskal–Wallis ANOVA: H (1) = 4.54, *p* = 0.03). The LC_50_ value for juveniles of slugs show the biggest concentration of toxin that kills 50% of individuals throughout the study (Table 2).

The survival of the adult slugs at low temperatures (Figure 1C,D) showed a larger decrease using lower concentrations of QE than in the first experiment (Figure 1A). The survival of slugs at 15 °C was significantly higher than the survival at 2 °C and −1 °C (Kruskal–Wallis ANOVA: H (1) = 4.07, *p* = 0.04 and H (1) = 6.84, *p* = 0.01, respectively). The LC_50_ for slugs at 2 °C and at −1 °C was half of the LC50 at 15 °C (Table 2). There was no significant difference between slug survival at 2 °C and −1 °C (Kruskal–Wallis ANOVA: H (1) = 0.39, *p* = 0.53). An increase in QE concentration from 20 to 50 g/L did not have a significant effect on the increase in adult slugs’ mortality (Figure 1C).

We observed moderate negative Spearman correlations between QE concentration and the survival of adult slugs at 2 °C (r_s_ = −0.44, *p* < 0.05; based on 255 slugs) and between QE concentration and survival of slugs at −1 °C (r_s_ = −0.51, *p* < 0.05; based on 170 slugs).

### 3.2. Wormicidal Activity of Saponins on E. albidus Worms

**Figure 2:**
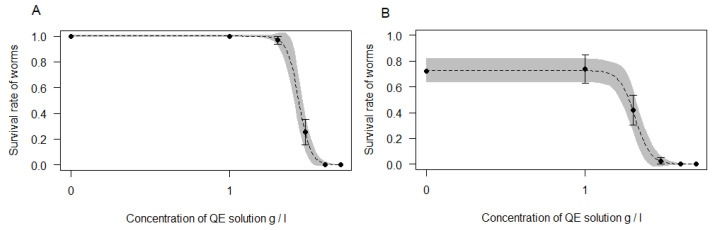
Dose–response curves for the toxicity of of *Quillaja saponaria* aqueous extract (QE) on: (**A**) *E. albidus* at 6 °C; (**B**) *E. albidus* at −1 °C. A grey band on the curve represents the standard error of the dose–response model. Black dots show the mean (with standard error bars) effect of the tested concentration of QE solution. Black dots represent tested concentrations of QE solution: 0; 1; 2; 3; 4; and 5 g/L.

The effect of saponins from QE on *E. albidus* exposed to five different doses for 14 days at 6 °C and −1 °C is illustrated in Figure 2. The initial concentration of QE of 1 and 2 g/L had no effect on the survival of worms at a favorable temperature (6 °C), but the concentration of 3 g/L immediately showed a lethal effect, with survival decreasing by 4.5 times and was only 25.4 ± 10%. The LC50 of QE on worms at 6 °C was 2.72 ± 0.09 g/L. The low temperature was characterized by a consistent lethal effect, which was enhanced by a saponins concentration of 2 g/L with survival that decreased to 42.1 ± 11.5%. The LC50 of QE on worms at −1 °C was 2.07 ± 0.11. When the worms were exposed to a concentration of 3 g/L, the population was practically destroyed. The survival was only 2.9 ± 2.5%. The LC50 of QE on *E. albidus* at 6 °C was several times lower than on slugs (Table 2).

We observed strong negative Spearman correlations between QE concentration and survival of *E. albidus* at 6 °C (r_s_ = −0.9, *p* < 0.05; based on 350 worms) and at −1 °C (r_s_ = −0.85, *p* < 0.05; based on 230 worms). This correlation was stronger than for slugs. In general, the survival of worms at 6 °C was significantly higher than survival at −1 °C (Kruskal–Wallis ANOVA: H (1) = 5.7, *p* = 0.02).

## 4. Discussion

Plant extracts containing tannins, saponins, and other substances have become the most interesting subject of molluscicidal research in recent decades [28]. There are many plant species rich in saponins, but their molluscicidal effects are different. Among these, *Q. saponaria*, *Camellia oleifera*, *C. sasanqua*, *Gleditsia amorphoides*, *Bidens pilosa*, *Saponaria officinalis*, *Pulsatilla chinensis*, and *Sapindus mukorossi* are the most investigated plant species that have molluscicidal properties [13,25,29,30,31,32,33].

Based on an overview of published data and our results (Table 3), we can conclude that QE saponins are not the strongest herbal extracts. González-cruz and Martín [13] tested *Deroceras reticulatum*, and Chaieb and Tayeb [31] tested *Theba pisana* with different herbal extracts and found that in order to obtain the same lethal effect, it was necessary to use higher doses of saponins from QE than from other plants. Our results have shown that even higher doses of saponins need to be used for the invasive slug *A. vulgaris*. Among the plant extracts containing other compounds, a stronger molluscicidal effect was shown by carvone from caraway seeds, *Carum carvi*. The LC_50_ was 0.75 mL/L (≈ 7.5 g/L) on *A. vulgaris* [34], but they used a methanol extract. Comparing these studies with our results, the molluscicidal effect of *Q. saponaria* aqueous extract was up to 12 times lower than the *C. carvi* methanol extract. We did not find estimated LC_50_ values for saponins to other soil animals, but Potter et al. [35] indicated that a dose of saponins-rich extract from *Camellia oleifera* seeds (131.5 g 1.5−2m) was deadly to earthworms (Lumbricidae) but did not affect black cutworms (*Agrotis ipsilon*), masked chafer (*Cyclocephala* sp.) grubs, Collembola, oribatid mites, mesostigmatid mites, aphids and midge (Chironomidae) larvae.

These studies suggest (Table 3) that the sensitivity of molluscs differs and depends on plant and mollusc species, individual age, and environmental conditions. For example, one of the most effective plants extracts for the control of freshwater snails *Oncomelania hupensis* is an aqueous extract from *Camelia sasanqua*. Meanwhile, for land molluscs, a more effective methanol extract comes from *Cestrum parqui* [31,33]. Moreover, it seems that freshwater snails are more sensitive to the toxicity of saponins than land snails (Table 3). The LC_50_ of saponin extracts on freshwater snails was less than on land snails and ranged from 0.001 g/L (aqueous extract) on *O. hupensis* [33] to ≈0.02 g/L (methanol extract) on *Pomacea canaliculata* [30]. For land snails, it ranged from 0.04 g/L (methanol extract) on *T. pisana* [31] to 96.94 g/L (aqueous extract) on *A. vulgaris* (our data). This can easily be explained by absorption through the mouth and the whole body surface; the water snail is dipped in water, while the terrestrial snail or slug absorbs through the sole or mouth. On the other hand, different herbal extracts were used. In the first case, extracts from *C. sasanqua* and *S. mucorossi* were tested, and in the second case, the extracts were from *C. parqui* and *Q. saponaria*. Methanol extracts of saponins have stronger molluscicidal effects. Moreover, it is possible that LC_50_ may vary according to the different observation time (Table 3).

Comparing the findings of de Souza et al. [25] with our results, the molluscicidal effect of QE aqueous extract on *A. vulgaris* adults does not stand out from the effect of *B. pillosa* aqueous extract on *Subulina octona* (Table 3). However, in both cases the plants were different and one case involved a slug of 70–150 mm in length, while *S. octona* is a much smaller snail with a 10–20 mm shell. The LC_50_ on *A. vulgaris* was 68.5 g/L, quite similar to the LC_50_ (≈51.4 g/L) on *S. octona* [25]. In both experiments, molluscs were exposed by dermal contact.

It cannot be excluded that the route of exposure of saponins extract into land molluscs can also have an important effect. The lethal effect of QE was about two times greater on *Deroceras* slugs after oral injections [13] than after exposure through dermal contact on *A. vulgaris* (our results, see Table 3). It should be noted that, in this case, slugs from different families were investigated.

Our research has revealed that the molluscicidal effect of saponins depends on the age of the molluscs and the temperature of the environment during the treatment. Contrary to the expectation that young slugs would be more sensitive to saponins, the results showed a completely opposite trend; the LC_50_ value at the same temperature (15 °C) was 1.7 times higher for *A. vulgaris* juveniles than adults. In contrast, most of the previous studies on snails [25,33] showed that molluscicidal extracts from plants were more lethal to younger and smaller snails than to adult and larger ones. This tendency has been explained by the bigger energy reserves of adult snails [25]. However, Anto et al. [37] and Sukumaran et al. [38] also found that the juvenile snails appeared less susceptible than the adults to the molluscicidal activity. The reason for this may be that the young stage slugs are highly active and hence more tolerant to different environmental stresses. This indicates that the stronger effect of molluscicides on juvenile or adult molluscs may vary from species to species and exposure type.

Both QE and freezing temperatures have similar sites of action considering that both are somehow affecting the membrane. Cold temperatures make membranes less fluid, and these become homeoviscous as adaptation response [39]. Lee et al. [40] stated that acclimation to the cold is often associated with an increase in membrane fluidity, which is directly associated with cell survival at low temperatures. The result that adult slugs are more sensitive to poisons at lower temperatures is important for countries with several generations of slugs. It would be insignificant in Eastern Europe as slugs have only one generation and adult slugs die naturally after laying eggs before autumn comes and before lower air temperatures appear [6]. However, these results suggest that, similar to adult slugs, juveniles also tend to be more sensitive to poisons at lower air temperatures and that they will be killed faster by using lower levels of poison in the autumn–winter time when they hatch from eggs.

There is a lack of research on how saponins affect soil organisms, and we expected its toxicity to other organisms to be lower than that of other plant extracts because, as reported above, the molluscicidal effect is not the strongest. However, some studies showed that saponins are capable of causing anti-feeding behavior in insects and to reduce their fertility [41]. Dowd et al. [42] exposed *Helicoverpa zea* and *Spodoptera frugiperda* caterpillars to 1000 ppm (≈1 g/L) *Q. saponaria* (20–35% by saponins content). After 10 days, the weight of the caterpillars was only 72.7% and 51.7% of control weight, respectively. This is a similar effective concentration of saponins as in the experiment with *E. albidus*. However, in the former experiment, the concentration was based on the diet whereas, in the latter experiment, it was based on dry weight of soil. Fischer et al. [36] showed that QE saponins had an LC_50_ of 150 ppm (≈0.15 g/L) on the plant parasitic nematode *Xiphinema index*. However, these experiments were conducted in a water environment.

The soil-dwelling enchytraeid, *E. albidus*, is a basic component of the soil ecosystem and more sensitive to compounds with amphiphilic characteristics (e.g., soaps, such as saponins) than other soil organisms, such as Collembola or predaceous mites [43]. Hence, enchytraeids are often used in eco-toxicological tests as sentinel species [21,44]. In this research experimental conditions for slugs and enchytraeids were not equal. There was a large difference between the average body volumes of the examined species, therefore enchytraeids got relatively bigger doses of saponins. In addition, whereas the slugs were exposed to saponins by lower body contact, the worms had it mixed to the soil where they were grown, likely affording a larger surface area of contact (and possibly uptake) with the saponins compared to the slug exposure method. It is also possible that the worm food supply could have been affected by the saponin, leading to an additional intake path besides body contact. Moreover, exposure time of slugs to saponins was two days, whereas that of worms was two weeks. The chosen methods were based on the possible usage of molluscicide: a solution of QE could be sprayed in areas with slugs which would come in contact with saponins through ground and plant surfaces. The solution could get deeper into the ground and affect soil animals (for example, worms). The LC_50_ at 15 °C on slugs was 68.5 g/L (adults) and 96.9 g/L (juveniles). The LC_50_ (at 6 ℃) on *E. albidus* was 2.72 g/L, far below those on *A. vulgaris*. Bearing in mind that the median effective doses for sub-lethal parameters (e.g., growth and reproduction) may well be one order of magnitude lower [43], it is evident that controlling slugs using saponins is likely to adversely affect enchytraeids, lumbricids, and possibly other soft-bodied invertebrates in treated sites.

## 5. Conclusions

The results of our studies show that *Q. saponaria* saponins may be a successful slug control tool used during the colder time of the year, but its concentration should be selected according to the age group of *A. vulgaris*. However, the unexpected toxicity to model organisms (white worms) limits its use as an environmentally benign alternative means of slug control.

## Figures and Tables

**Table 1:** List of the tested groups and the main conditions of experiments

Species of Groups	Number of Individual	Temperature (°C)	Weight of Animal, Av ± SE (g)	Duration of Exposure (Days)
*A. vulgaris* ad.	125	+15	8.3 ± 2.5	2
*A. vulgaris* ad.	255	+2	8.4 ± 0.3	2
*A. vulgaris* ad.	170	−1	8.7 ± 0.3	2
*A. vulgaris* juv.	125	+15	0.05 ± 0.01	2
*E. albidus*	350	+6	0.04 ± 0.001	14
*E. albidus*	230	−1	0.04 ± 0.001	14

Av = average, SE = standard error.

**Table 2:** Dose–response parameters of *Quillaja* aqueous extract for slugs and worms in g/L (average ± standard error).

Species	Life Stage	Temperature (°C)	LC_50_	Slope	χ^2^ (df, *p*)
*A. vulgaris*	Adult	+15	68.52 ± 8.38	1.51 ± 0.29	15.68 (27, 0.9588)
*A. vulgaris*	Juvenile	+15	96.94 ± 13.84	1.71 ± 0.4	16.731 (17, 0.4727)
*A. vulgaris*	Adult	+2	33.52 ± 7.49	0.98 ± 0.31	29.077 (30, 0.5135)
*A. vulgaris*	Adult	−1	18.38 ± 3.64	1.06 ± 0.3	20.986 (31, 0.9123)
*E. albidus*	Adult	+6	2.72 ± 0.09	11.85 ± 2.45	22.792 (29, 0.7857)
*E. albidus*	Adult	−1	2.07 ± 0.11	9.09 ± 2.61	51.252 (46, 0.2753)

LC_50_—median lethal concentration, χ^2^—goodness of fit of probit lines.

**Table 3:** Summary data for the toxicity of aqueous (or methanol) saponin extract from different plants for molluscs and worms.

Species and Age Group	Temperature (°C)	Duration of Exposure (Days)	Plant Aqueous (or Methanol) Extract	LC_50_ (g/L)	Reference
**Molluscs**					
*Arion vulgaris*, ad.	+15	2	*Quillaja saponaria*	68.52	Present study
	+2	2		33.52	
	−1	2		18.38	
*A. vulgaris* juv.	+15	2	*Q. saponaria*	96.94	Present study
*Deroceras*	+15	5	*Q. saponaria*	40	[13]
*reticulatum*, ad.	+15	5	*Gleditchia amorphoides*	≈20	
	+15	5	*Camelia oleifera*	≈20	
*Subulina octona*, ad.	+21–24	2	*Bidens pilosa*	≈51.4	[25]
*Theba pisana*, ad.	+21–24	1	*Q. saponaria*	≈0.54	[31]
	+21–24	1	*Cestrum parqui* *	≈0.04	
*T. pisana*, juv.	+21–24	1	*Q. saponaria*	≈0.29	
	+21–24	1	*C. parqui* *	≈0.01	
*Pomacea canaliculata,* ad.	+21–24	2	*Sapindus mucorossi* *	≈0.02	[30]
*Oncomelania hupensis,* ad.	+25	1	*Pulsatilla chinensis*	≈0.001	[32]
	+21–24	2	*Camelia sasanqua*	≈0.002	[33]
**Worms**					
*Enchytraeus albidus*	+6	14	*Q. saponaria*	2.84	Present study
(Olygochaeta), ad.	−1	14		2.16	
*Xyphinema index*	+22	3	*Q. saponaria*	≈0.15	[36]
(Nematode), ad.					

*—methanol extract, LC50—median lethal concentration of toxin, ad.—adult, juv.—juvenile
